# Pantothenate Kinase 1 Is Required to Support the Metabolic Transition from the Fed to the Fasted State

**DOI:** 10.1371/journal.pone.0011107

**Published:** 2010-06-14

**Authors:** Roberta Leonardi, Jerold E. Rehg, Charles O. Rock, Suzanne Jackowski

**Affiliations:** 1 Department of Infectious Diseases, St. Jude Children's Research Hospital, Memphis, Tennessee, United States of America; 2 Department of Pathology, St. Jude Children's Research Hospital, Memphis, Tennessee, United States of America; University of Florida, United States of America

## Abstract

Coenzyme A (CoA) biosynthesis is regulated by the pantothenate kinases (PanK), of which there are four active isoforms. The PanK1 isoform is selectively expressed in liver and accounted for 40% of the total PanK activity in this organ. CoA synthesis was limited using a *Pank1* ^−/−^ knockout mouse model to determine whether the regulation of CoA levels was critical to liver function. The elimination of PanK1 reduced hepatic CoA levels, and fasting triggered a substantial increase in total hepatic CoA in both *Pank1* ^−/−^ and wild-type mice. The increase in hepatic CoA during fasting was blunted in the *Pank1* ^−/−^ mouse, and resulted in reduced fatty acid oxidation as evidenced by abnormally high accumulation of long-chain acyl-CoAs, acyl-carnitines, and triglycerides in the form of lipid droplets. The *Pank1* ^−/−^ mice became hypoglycemic during a fast due to impaired gluconeogenesis, although ketogenesis was normal. These data illustrate the importance of PanK1 and elevated liver CoA levels during fasting to support the metabolic transition from glucose utilization and fatty acid synthesis to gluconeogenesis and fatty acid oxidation. The findings also suggest that PanK1 may be a suitable target for therapeutic intervention in metabolic disorders that feature hyperglycemia and hypertriglyceridemia.

## Introduction

Coenzyme A (CoA) is an essential cofactor involved in energy metabolism that carries carboxylic acid substrates and supports a multitude of oxidative and synthetic metabolic reactions, including those involved in the citric acid cycle, sterol, bile acid and fatty acid biosynthesis, and fatty acid oxidation (for review, see [1]). CoA is derived from vitamin B_5_ (pantothenate), cysteine and ATP. Pantothenate kinase (PanK) catalyzes the first committed step and controls the overall rate of CoA biosynthesis [1]–[3]. In mammals, there are three genes that express four characterized isoforms of PanK. PanK1α and PanK1β are encoded by the same gene and arise from the use of alternate initiation exons [4], whereas the PanK2 and PanK3 isoforms are encoded by distinct genes [5], [6]. The PanK isoforms are feedback regulated by CoA thioesters [4], [5], [7], and the loss or alteration of feedback regulation by mutations in PanK result in dysregulated CoA production [5], [8]. Thus, PanK expression levels contribute to determination of the cellular CoA content [9], [10].

The multiplicity of PanK isoforms suggests tissue-specific CoA regulation. PanK1 transcripts are most highly expressed in liver. PanK2 is most abundant in neuronal tissue and PanK3 is widely and highly expressed in all tissues examined [6], [10]. The importance of CoA biosynthesis in metabolism is clearly evident from a chemical knockout of CoA synthesis at the PanK step using a pantothenate antimetabolite which targeted all PanK isoforms [11]. Mice treated with the inhibitor exhibited a selective and dramatic reduction in CoA levels in liver and kidney that resulted in significant changes in mitochondrial morphology, defective fatty acid oxidation, and hypoglycemia. Metabolic and gene expression profiling revealed the critical function of unesterified CoA in supporting gluconeogenesis and fatty acid oxidation [11].

The physiological adaptations associated with the transition from the fed to the fasted state have been the focus of considerable experimental interest [12]. The liver is central to the body's response to nutrient deprivation and switches from an organ of glucose oxidation and fatty acid synthesis in the fed state to one of glucose and ketone body production and fatty acid oxidation in the fasted state [13]–[15]. Transcriptional regulators like PGC1α and PPARα are activated during a fast and drive a genetic program that alters the levels of key enzymes and regulatory factors in intermediary metabolism, including the enzymes of fatty acid oxidation [16]. The importance of fatty acid oxidation during fasting to provide energy for gluconeogenesis is evident from the study of inborn errors in mitochondrial β-oxidation and of knockout mice with deficiencies in individual β-oxidation enzymes [17]. CoA is a required cofactor for β-oxidation and a significant increase in liver CoA is associated with the transition from the fed to the fasted state [18]–[22]. It is not known whether elevated intracellular CoA concentrations are as important to supporting β-oxidation during fasting as the increased activities of fatty acid β-oxidation enzymes.

The PanK1 isoform is highly and selectively expressed in liver, thus we derived a *Pank1* ^−/−^ mouse model to elucidate the role of this regulatory enzyme in the CoA response to fasting. The PanK1-deficient liver had lower CoA, and during a fast, fatty acid oxidation was impaired resulting in the accumulation of long-chain acyl-CoAs, acyl-carnitines and triglycerides as lipid droplets. Gluconeogenesis in the fasted state was profoundly impaired in the *Pank1* ^−/−^ mice, resulting in hypoglycemia. Ketogenesis was unimpaired. These results illustrate that increasing the supply of CoA is just as important as the expression/activity of the β-oxidation enzymes for fatty acid metabolism during adaptation to nutrient deprivation.

## Results

### Derivation and General Characteristics of the Pank1 Knockout Mice

**Figure 1:**
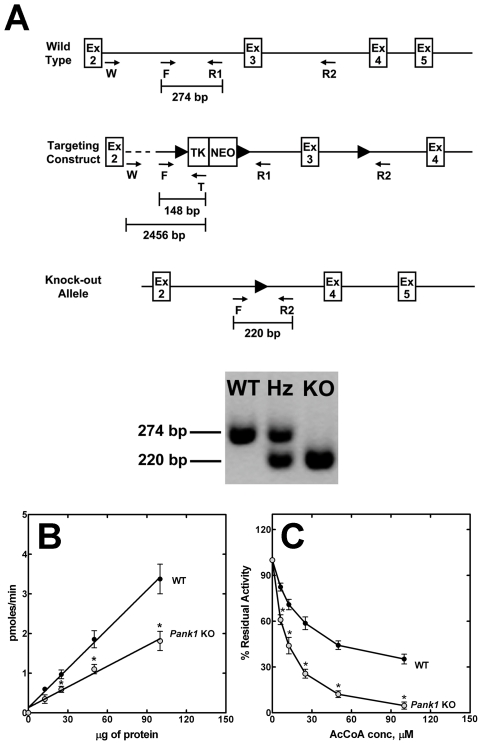
Generation of *Pank1* ^−/−^ mice and liver PanK activity. (**A**) Diagram of the gene targeting strategy. A selection cassette containing cDNAs encoding thymidine kinase (TK) and neomycin resistance (NEO) was inserted into the intron between exons 2 (Ex2) and 3 (Ex3) of the *Pank1* gene. The cassette was flanked by loxP (▸) sites. A third loxP site was inserted between exons 3 (Ex3) and 4 (Ex4). Cre-recombinase-mediated deletion of the DNA between the outermost loxP sites resulted in a knockout (KO) allele which lacked Ex3 and the selection cassette. Locations of the primers (arrows) for PCR analysis and genotyping are shown. PCR products and sizes are indicated (bars). Locations of the loxP sites are indicated with triangles. A PCR product of 274 bp using primers F and R1 indicated a wild-type (WT) allele, and a PCR product of 220 bp using primers F and R2 indicated a deleted (KO) allele. Heterozygous (Hz) mice had both alleles. (**B**) Total PanK activity (pmoles/min) was determined in liver homogenates from wild-type (WT; •) or *Pank1* knockout (KO; ◯) mice as a function of protein concentration. The data are the average of duplicate measurements obtained from 4 control and 5 knockout mice. (**C**) Inhibition of total PanK activity by acetyl-CoA (AcCoA) in liver homogenates from wild-type (WT; •) or *Pank1* knockout (KO; ◯) mice. For WT, the 100% value corresponds to 33±3 pmoles/min/mg protein, and 17±2 pmoles/min/mg protein for KO. The data are the average of duplicate measurements obtained with 3 mice of each genotype. Statistically significant differences between wild-type and knockout are indicated by * (p<0.05).

Recombinant embryonic stem cells were obtained and mice were generated that were heterozygous for a *Pank1* gene that was modified by insertion of loxP sites in the introns flanking exon 3 (Fig. 1A). Mating with an ACTB-Cre transgenic mouse strain resulted in pups with a *Pank1* allele in which exon 3 was deleted via Cre recombinase-dependent excision of the DNA between the first and third loxP sites in all tissues early in embryogenesis. The deletion resulted also in the removal of the cDNA cassette used for selection of the recombinant embryonic stem cells. *Pank1* ^+/−^ heterozygous mice were mated and pups with a homozygous deletion of the *Pank1* gene were obtained in the expected Mendelian ratio as determined by tail genotyping (Fig. 1A). The ACTB-Cre transgene was not maintained in the knockout mouse line. The experiments were performed with 12–16 week old male *Pank1* ^−/−^ mice.

The *Pank1* knockout mice did not exhibit gross defects in anatomy or behavior. The *Pank1* ^−/−^ mice weighed more at 10 weeks and were significantly heavier at 16 weeks compared to the control littermates (Table 1). Serum parameters were the same comparing the control and knockout mice with the exception of blood glucose and triglycerides, which were lower in the knockout mice after an overnight fast (Table 1). There was no elevation in serum transaminases and no evidence of liver damage or inflammation. Although PanK1 was expressed in kidney [10], there was no indication of a defect in kidney function from the serum and urine analysis. Neither the wild-type nor the *Pank1* ^−/−^ knockout mice had detectable urobilinogen, glucose, ketones, bilirubin, or blood in the urine (not shown). Heart is the only other tissue expressing *Pank1* [10], but real-time qRT-PCR measurements showed that heart *Pank1* was expressed at less than 2% the level of *Pank1* in the liver. PanK3 was the major isoform in heart (data not shown). Thus, the investigation of the PanK1-null phenotype focused on CoA-mediated metabolism in liver.

**Table 1:** General features of the *Pank1* ^−/−^ mice.

Parameter	Mouse Genotype	
	Wild-Type	*Pank1* ^−/−^
Weight, g, 10 weeks	24.7±0.6 (11)	26.7±0.7 (16)
Weight, g, 16 weeks	27.0±0.8 (11)	30.2±0.9 (15)*
Glucose, mg/dL	74±2 (24)	55±2 (26)*
Cholesterol, mg/dL	121±9 (6)	111±13 (6)
Triglycerides, mg/dL	160±9 (8)	113±9 (7)*
Free Fatty Acids, mM	1.3±0.1 (5)	1.2±0.2 (5)
AST^a^, U/L	172±39 (6)	181±39 (6)
ALT^b^, U/L	81±25 (6)	76±17 (6)
Ketones (fed), mM	0.23±0.02 (9)	0.28±0.05 (9)
Ketones (48h fast), mM	1.8±0.32 (13)	2.5±0.56 (11)

Animals were fasted overnight (14 h) prior to the serum measurements. Values are reported as the mean ± standard error (number of mice).

a Aspartate aminotransferase.

b Alanine aminotransferase.

*p<0.05.

### Liver PanK Activity

The *Pank1β* transcripts accounted for 58% of the total PanK mRNAs in wild-type liver (Table 2). Only 2% of the PanK1 isoform mRNAs were present as *Pank1α*, and *Pank2* and *Pank3* accounted for 7% and 33% of the total PanK transcripts, respectively. The expression of the *Pank2* or *Pank3* genes did not increase in the PanK1-deficient liver to compensate for loss of *Pank1* expression (Table 2). There was a 40% decrease in the total PanK enzyme specific activity in knockout liver extracts (Fig. 1B). This assay used unfractionated extracts and measured the sum of all PanK isoforms. The decrease in total PanK activity in the *Pank1* ^−/−^ extracts was consistent with the loss of *Pank1* expression. A distinguishing feature of PanK1β is that it is less sensitive to acetyl-CoA inhibition than the other PanK isoforms [4], [5], [7]. Accordingly, extracts from wild-type livers possessed a fraction of the PanK activity that was refractory to acetyl-CoA inhibition (Fig. 1C). The acetyl-CoA-insensitive PanK activity that constituted about 40% of the total PanK activity in the wild-type liver extract was absent from liver extracts obtained from *Pank1* ^−/−^ mice (Fig. 1C). Thus, the PanK2 and PanK3 isoforms accounted for all of the PanK activity in the livers from *Pank1* ^−/−^ mice.

**Table 2:** Effect of fasting on the mRNA of the PanK and CoA diphosphatase (nudix hydrolase) isoforms in the livers of wild-type and *Pank1^−/−^* mice.

	Relative *Pank* and *Nudt* isoform mRNA abundance in liver					
Gene	Fed		24 h Fasted		48 h Fasted	
	Wild-Type	*Pank1^−/−^*	Wild-Type	*Pank1^−/−^*	Wild-Type	*Pank1^−/−^*
*Pank1α*	0.25±0.09 (5)	ND (5)	0.26±0.08 (4)	ND^a^(5)	0.35±0.17 (9)	ND (7)
*Pank1β*	10.00±2.13 (5)	ND (5)	12.62±2.42 (5)	ND (5)	13.76±5.80 (9)	ND (7)
*Pank2*	0.95±0.18 (5)	0.83±0.20 (5)	0.84±0.09 (5)	0.79±0.17 (5)	1.08±0.24 (9)	0.40±0.12^c^(7)
*Pank3*	5.41±1.15 (5)	5.94±1.51 (5)	5.06±1.21 (5)	5.47±1.10 (5)	6.03±1.29 (9)	3.2±1.42^c^(7)
*Nudt7*	2.11±0.62 (5)	2.77±0.75 (7)	NA^b^	NA	0.77±0.34^d^(7)	0.63±0.39^d^(7)
*Nudt19*	0.016±0.003 (5)	0.033±0.018 (7)	NA	NA	0.079±0.034^d^(7)	0.051±0.017 (7)

The PanK1β transcript was the most abundant PanK mRNA in the wild type liver, and its level was set at 10. The abundance of all other PanK isoforms was expressed relative to PanK1β. The mRNA abundance of nudix hydrolases is reported relative to a cyclophilin calibrator. Each transcript was measured in triplicate for each mouse sample, and the combined results are shown, ± s.d. (number of mice).

a ND means not detectable.

b NA means not analyzed.

c Means statistically significant difference between *Pank1^−/−^* and controls (p<0.05).

d Means statistically significant difference between fed and fasted (p<0.05).

### Food Consumption

**Figure 2:**
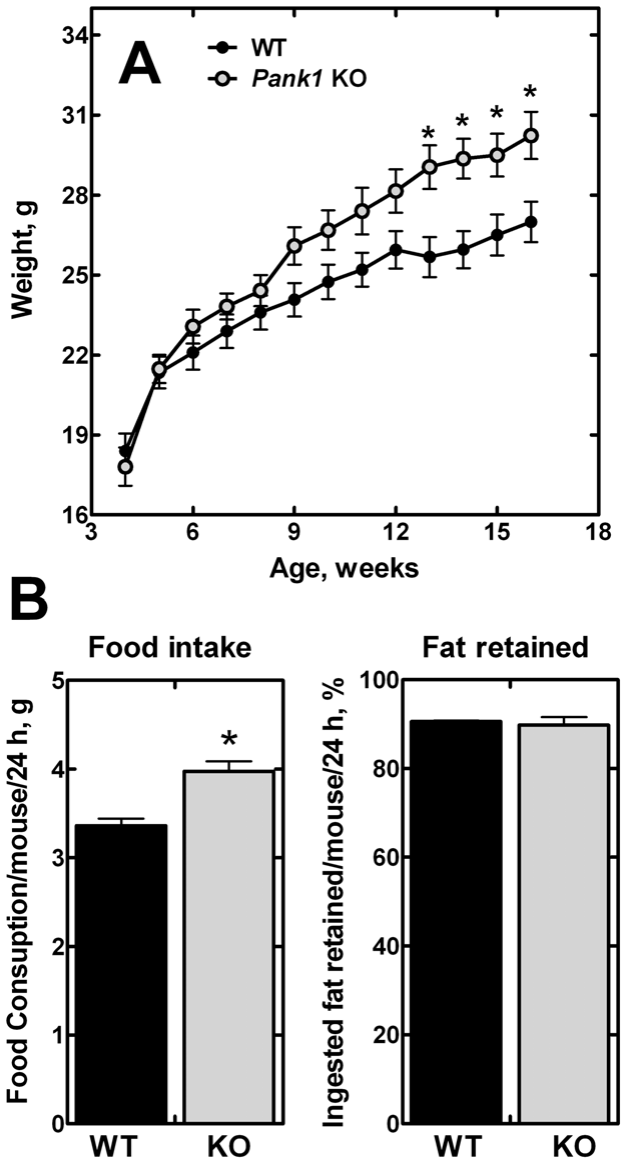
Weight monitoring and food intake in *Pank1 knockout* mice. (**A**) Body weight as a function of age was measured in 11 wild-type (WT) and 16 knockout (KO) mice. (**B**) Food intake: food consumption was monitored in 16 wild-type (WT) and 11 knockout (KO) mice. Fat retained: the percentage of fat retained by 4 wild-type (WT) and 8 knockout (KO) mice. 100% corresponds to 200 mg of lipids retained/mouse/24 hours for WT, and 276 mg of lipids retained/mouse/24 hours for KO. These data were calculated from the measured amount of lipid ingested minus the measured amount of lipid excreted in the feces. Statistically significant differences between wild-type and knockout are indicated by * (p<0.05).

The higher weight noted for the *Pank1* ^−/−^ mice (Table 2) led to a more comprehensive study. Weight monitoring clearly showed the *Pank1* ^−/−^ mice gained more weight as they aged compared to their littermate controls (Fig. 2A). A food monitoring study revealed that the *Pank1* ^−/−^ mice ate significantly more food than the controls, and retained the same percentage of the fat calories as the control animals (Fig. 2B). These data revealed that hyperphagy accounted for the greater weight gain of the *Pank1* ^−/−^ mice. CoA metabolism in the hypothalamus has been implicated in the regulation of feeding behavior [23], and so we determined the relative distribution of the PanK isoforms in wild-type hypothalami. The Pank1 transcripts constituted 6.2% (n = 3 mice) of the PanK isoforms, and thus a perturbation of CoA metabolism may underly the increased food consumption.

### Reduced CoA Levels and Fatty Acid Oxidation

**Figure 3:**
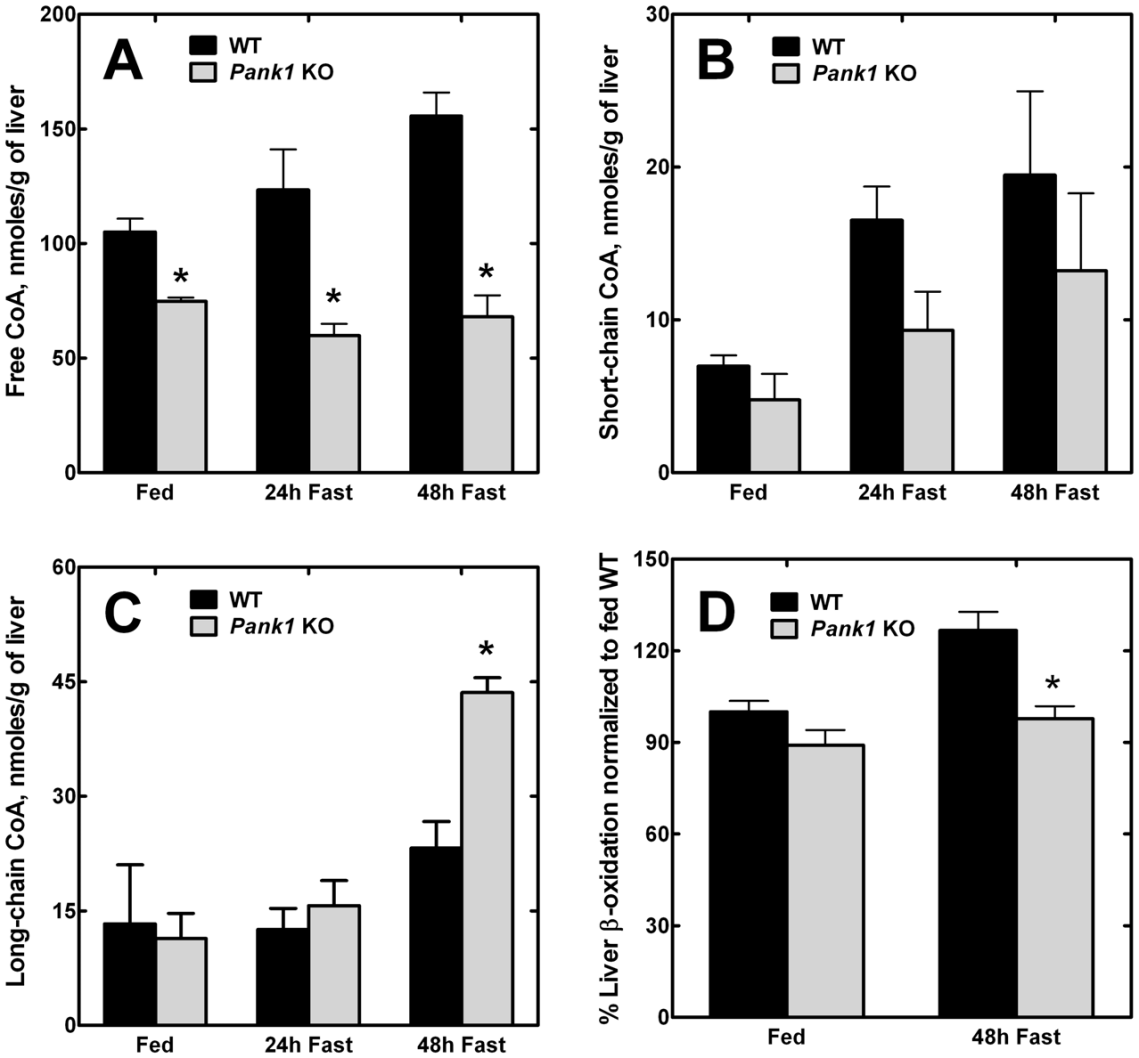
CoA levels and fatty acid oxidation in the PanK1-deficient liver. (**A**) Levels of free CoA were measured in livers from 4 wild-type (WT) and 4 *Pank1* knockout (KO) mice and reported as a function of tissue wet weight. The mice were fed ad libitum, or fasted for either 24 or 48 h. The data are the averages of duplicate measurements on each mouse of each genotype ± standard error. (**B**) Levels of short-chain acyl-CoA were measured in livers from wild-type (WT) and *Pank1* knockout (KO) mice and reported as a function of tissue wet weight. The acyl chain lengths include C2 through C12, inclusive. The mice were fed ad libitum, or fasted for either 24 or 48 h. The data are the averages from duplicate measurements from 4 control and 4 knockout mice ± standard error. (**C**) Levels of long-chain acyl-CoA were measured in livers from wild-type (WT) and Pank1 knockout (KO) mice and reported as a function of tissue wet weight. The acid-insoluble long-chain acyl-CoAs include C14∶0 and longer [75]. The data are the averages from duplicate measurements from 4 control and 4 knockout mice ± standard error. (**D**) Rates of fatty acid oxidation were measured in liver extracts derived from 4 fed wild-type (WT) or 5 fed knockout (KO) mice. Rates of fatty acid oxidation were measured in liver extracts derived from 4 wild-type (WT) or 5 knockout (KO) mice fasted for 48 h. 100% corresponds to 7.7 pmoles of ^3^H-palmitic acid oxidized/min/mg protein. The data are the averages of duplicate measurements from control and knockout mice ± standard error. Statistically significant differences between wild-type and knockout are indicated by * (p<0.05).

We measured hepatic CoA levels during fasting and compared mice fed regular chow *ad libitum* and mice fasted for 24 or 48 h (Fig. 3). These data show that the free CoA pool is the largest CoA species in mammalian cells, in agreement with our previous measurements [11]. Note the difference in scale between the free CoA measurements compared to the short-chain and long-chain acyl-CoAs. There was a significant increase in the free and short-chain acyl CoA content following fasting of the wild-type animals that reached the highest levels at 48 h (p<0.05) (Fig. 3A and 3B). In contrast, the PanK1-deficient livers had lower free CoA than wild-type livers under both fed and fasted conditions (p<0.05), and the free CoA did not increase during fasting in the knockouts (Fig. 3A). Meanwhile, short-chain acyl-CoA levels increased modestly in the knockout animals during a 48 h fast. Although the data indicated a lower short-chain acyl-CoA trend in the knockouts, the levels were not significantly different from those of the wild-type animals (Fig. 3B). The overall increase in CoA content during fasting was not driven by increases in the transcript levels of the PanK genes (Table 2). In wild-type animals, none of the PanK genes exhibited increased expression during fasting, and the elimination of PanK1 expression did not increase the transcript levels for *Pank2* or *Pank3* following fasting. In fact, there was a decrease in the level of *Pank2* and *Pank3* mRNAs in the fasted, knockout animals. On the other hand, the expression of mouse nudix hydrolase 7 (Nudt7), a liver-specific CoA-phosphodiesterase [24], was downregulated during fasting in both wild-type and knockout livers (Table 2), in agreement with previous results [25], [26]. The Nudt19 CoA phosphodiesterase [27] was also expressed, but the contribution of the Nudt19 isozyme was quantitatively 10-fold less than Nudt7, based on the transcript levels after fasting. These data were consistent with decreased CoA degradation during the fasting response, thus contributing to the increase in free CoA in the wild-type livers and the increased short-chain acyl-CoAs in both control and PanK1-deficient livers (Fig. 3A).

A significant difference between wild-type and knockout animals was the marked increase in long-chain acyl-CoAs in the *Pank1* ^−/−^ livers after a 48 h fast (Fig 3C). While the long-chain acyl-CoA levels in wild-type livers increased during fasting, as expected, the levels in the knockout livers were abnormally high. These data pointed to a defect in long-chain acyl-CoA metabolism. Fifteen minute measurements of the rates of fatty acid oxidation demonstrated reduced capacity for oxidation in the fasted *Pank1* ^−/−^ livers compared to fasted wild-type livers (Fig. 3D). The fatty acid oxidation capacity increased substantially in the wild-type livers after a 48h fast, but the capacity of the knockout livers remained the same as in the fed state. The oxidation measurements were made without the addition of CoA, and thus reflected the endogenous free CoA as well as the changes in gene expression engendered by fasting, largely mediated by activation of PPARα. Thus, the increase in long-chain acyl-CoAs (Fig. 3C) was consistent with the decreased capacity for acyl-CoA oxidation during fasting when the animals became more reliant on mobilized fatty acids as an energy source.

**Figure 4:**
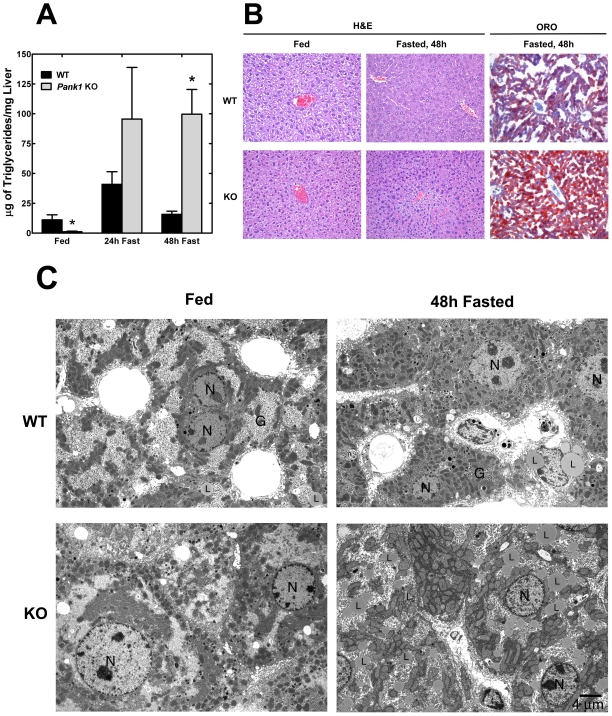
Development of a fatty liver in fasted *Pank1* ^−/−^ mice. (**A**) TG levels in 4 wild-type (WT) and 4 PanK1 knockout (KO) mouse livers were determined in the fed state and following a 24 or 48 h fast. Statistically significant differences between wild-type and knockout values are indicated by * (p<0.05). (**B**) Histology of wild-type (WT) and PanK1 knockout (KO) liver in the fed state and following a 24 or 48 h fast. Liver tissue was stained with either hemotoxylin-eosin (H&E) or oil red O (ORO). (**C**) Transmission electron microscopy of hepatocytes from fed and 48-h fasted wild-type (WT) and *Pank1* knockout (KO) mice. N, nucleus; G, glycogen, and L, lipid droplets. Statistically significant differences between wild-type and knockout samples are indicated by * (p<0.05).

Analysis of the liver lipid composition corroborated this view (Fig. 4A). In control mice, liver triglyceride levels increased slightly after a 24 h fast and then returned to the fed level at 48 h. In contrast, the PanK1-deficient livers actually had lower levels of triglycerides than the control mice in the fed state, and the triglycerides also increased after a 24 h fast. The amount of hepatic triglyceride was variable at 24 h, with some *Pank1* ^−/−^ animals having levels similar to wild-type while others had significantly elevated triglycerides, giving rise to a large spread in the values at this time point. However, by 48 h, the triglyceride content of the CoA-deficient livers was consistently 5 times higher than in the wild-type controls (Fig. 4A). The storage of excess fatty acid as triglyceride in the knockout livers illustrated that the amount of free CoA became limiting to fatty acid oxidation in the physiologic context of the fasted state, when there is increased fatty acid mobilization and an upregulation of fatty acid degradative gene expression mediated by PPARα activation.

### Histology of the *Pank1*-Deficient Liver

Histological examination of liver sections did not reveal any marked differences between the control and *Pank1* ^−/−^ mice in the fed state (Fig. 4B). Following a 48 h fast, numerous cellular inclusions were noted in the PanK1-deficient livers, and staining with oil red O clearly showed the accumulation of neutral lipid droplets (Fig. 4B). This finding was corroborated by electron microscopy (Fig. 4C). Occasional lipid droplets were observed in the wild-type fed livers and similarly after a 48 h fast. In contrast, lipid droplets were rare in the fed CoA-deficient livers and the hepatocytes became replete with lipid droplets after a 48 h fast. These data were in agreement with the triglyceride measurements (Fig. 4A), and supported a defect in the catabolism of fatty acids in the fasted CoA-deficient livers.

### Accumulation of Long-chain Acyl-Carnitines

**Figure 5:**
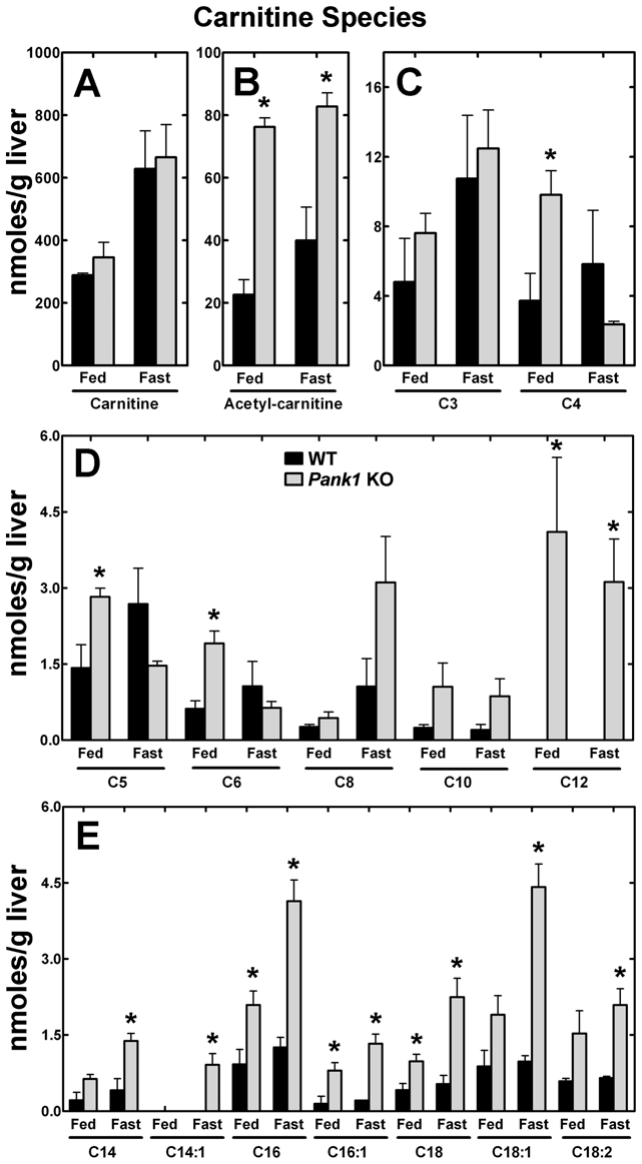
Elevated long-chain acyl-carnitines in the fasted *Pank1* knockout liver. Acyl-carnitines were extracted from the livers of 3 wild-type (WT) and 3 *Pank1* knockout (KO) mice that were fasted for 48 h and each liver was extracted and quantified by mass spectrometry as described in “Experimental Procedures.” (**A**) Free carnitine levels. (**B**) Acetyl-carnitine levels. (**C**) Propanoyl- and butanoyl-carnitine levels. (**D**) Medium-chain acyl-carnitine levels. (**E**) Long-chain acyl-carnitine levels. Statistically significant differences between wild-type and knockout values are indicated by * (p<0.05).

The acyl-carnitine levels in the wild-type and CoA-deficient livers were determined in the fed state and following a 48 h fast for two reasons. First, long-chain acyl-carnitines are activators of PanK2 [7] and PanK3 (Leonardi and Jackowski, unpublished data). Allosteric activation of these PanKs by acyl-carnitine could contribute to the increase in CoA levels in the fasted liver. Second, acyl-carnitine levels are an indicator of the efficiency of fatty acid oxidation and accumulate in cells and body fluids when fatty acid oxidation is impaired [28], [29]. Free carnitine was the most abundant intracellular species and increased substantially following fasting and to the same extent in both control and PanK1-deficient livers (Fig. 5A). Carnitine synthesis and uptake are both stimulated by activation of PPARα [30] and these measurements reflect a normal fasting response in both control and knockout animals. Acetyl-carnitine was significantly higher in the CoA-deficient livers in both the fed and fasted states (Fig. 5B). The transfer of acetyl moieties between carnitine and CoA occurs in mitochondria and reflected the CoA deficiency and the importance of liberating nonesterified CoA to support fatty acid degradation [11]. There were only minor differences in the 3- and 4-carbon acyl-carnitines (Fig. 5C). A striking accumulation of >C10 acyl-carnitines in the PanK1-deficient livers was evident in both the fed and fasted states, suggesting that activation of PanK2 and/or PanK3 may contribute to supporting tissue CoA levels in the absence of PanK1 (Fig. 5D and 5E). The amounts of C16∶0, C18∶0 and C18∶1 acyl-carnitines increased significantly following a 48 h fast, reflecting the reduced capacity for fatty acid oxidation in the CoA-deficient hepatocytes. In contrast, the >C10 acyl-carnitines were lower in the wild-type livers and the distribution did not change significantly after fasting, indicating that long-chain acyl-carnitine activation of PanK2/3 did not contribute to the increased CoA during fasting in the wild-type liver. These metabolomic measurements supported the conclusion that there was impaired fatty acid degradation in the CoA-deficient hepatocytes.

**Figure 6:**
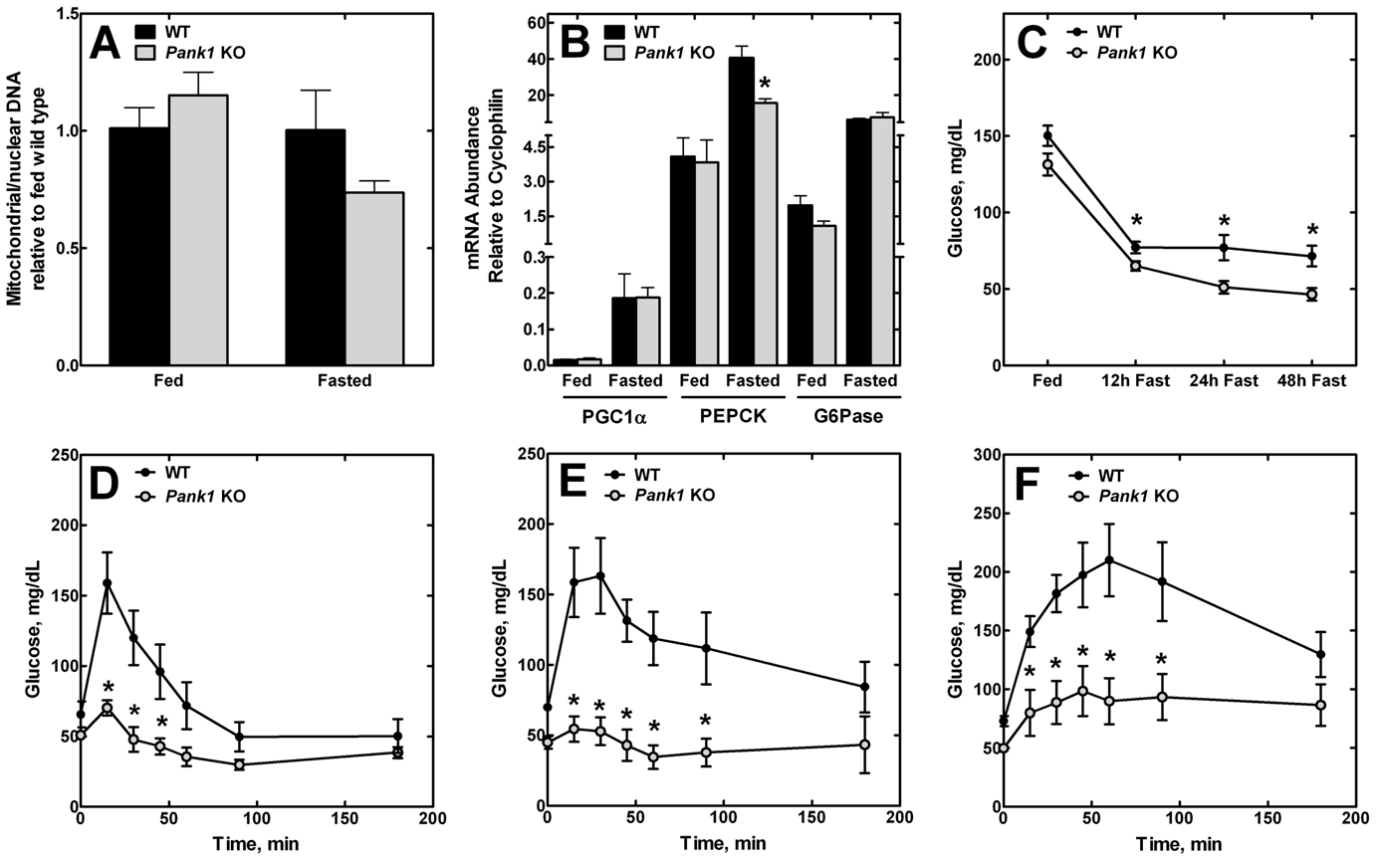
Liver mitochondrial number and global glucose homeostasis in the *Pank1* knockout mice. (**A**) The number of mitochondria per cell was estimated using quantitative real-time PCR to measure the ratio of mitochondrial to nuclear DNA in livers from 4 wild-type (WT) and 5 *Pank1* knockout (KO) mice each in the fed state and after a 48 h fast. (**B**) qRT-PCR quantification of PGC1α, phosphoenoylpyruvate carboxykinase (PEPCK) and glucose-6-phosphatase (G6Pase) mRNA in the livers of fed or fasted wild-type (WT; n = 5) and *Pank1* knockout (KO; n = 5) mice. (**C**) Serum glucose levels wild-type (WT; n = 9) and *Pank1* knockout (KO; n = 10) mice during fasting. (**D**) Gluconeogenesis in 4 wild-type (WT) and 4 *Pank1* knockout (KO) mice measured with a pyruvate challenge test. (**E**) Gluconeogenesis in 6 wild-type (WT) and 6 *Pank1* knockout (KO) mice measured with an oxaloacetate challenge test. (F) Gluconeogenesis in 9 wild-type (WT) and 10 *Pank1* knockout (KO) mice measured with a glycerol challenge test *, p<0.05.

Fatty acid oxidation occurs in mitochondria and peroxisomes, which sequester a substantial fraction of total cellular CoA [1]. Mitochondria have an active CoA transport mechanism [31], [32] and achieve millimolar concentrations within the matrix [33], [34]. CoA plays a major role as an acyl group carrier in these pathways and the availability of unesterified CoA is critical to the operation of fatty acid oxidation, tricarboxylic acid metabolism and the urea cycle [11]. The lower levels of total CoA in the *Pank1* ^−/−^ liver suggested there was either a reduced amount of CoA per mitochondrion or fewer mitochondria in the *Pank1* ^−/−^ mice. A quantitative measurement of the nuclear to mitochondrial DNA copy number was performed to determine if there was a difference between the knockout and wild-type mice during the fasting response. We did not find a significant difference in the number of mitochondria in the control or CoA-deficient livers, fed or fasted (Fig. 6A). These data indicated that the intracellular CoA concentration did not impact mitochondrial biogenesis, but rather a deficiency in the amount of CoA per mitochondrion contributed to the global reduction in fatty acid oxidation in the fasted livers of the knockout mice.

PGC1α is induced during fasting and is a major contributor to the induction of gluconeogenesis. The levels of this factor were elevated 10–12 fold and there was no difference between the control and *Pank1* ^−/−^ livers (Fig. 6B). Also, the elevations in phosphoenoylpyruvate carboxykinase (4–10 fold) and glucose-6-phosphatase (3–8 fold) mRNAs that are normally associated with fasting occurred in control and *Pank1* ^−/−^ livers (Fig. 6B). These data illustrate that the genetic program that switches the liver to gluconeogenesis during fasting was intact in the CoA-deficient livers.

### Glucose Homeostasis in *Pank1^−/−^* Mice

Analyses of 14-hour fasted serum glucose levels indicated that the *Pank1* ^−/−^ mice had lower serum glucose than the wild-type controls (Table 1). Therefore, we examined glucose levels as a function of time after initiating a fast (Fig. 6C). The control mice responded with depressed blood glucose during the fast (Fig. 6C). The *Pank1* ^−/−^ mice had significantly lower blood glucose levels at each time point.

Despite a normal induction of the gluconeogenic genes (Fig. 6B), the data suggested that the *Pank1* ^−/−^ mice became progressively more compromised in gluconeogenesis as the fast progressed. A direct test of this idea was a pyruvate challenge experiment that measured blood glucose in response to the administration of pyruvate, a major gluconeogenic substrate (Fig. 6D). The *Pank1* ^−/−^ mice were severely compromised in the pyruvate challenge. We reasoned that pyruvate carboxylase, the first step in the gluconeogenic pathway from pyruvate, may have been affected by CoA depletion because it is a mitochondrial enzyme that absolutely requires acetyl-CoA as a cofactor [35]. This idea was tested with an oxaloacetate challenge and a glycerol challenge (Fig. 6E). Oxaloacetate and glycerol are cytosolic gluconeogenic intermediates that enter the pathway downstream of pyruvate carboxylase. Gluconeogenesis from these substrates would be independent of pyruvate carboxylase activity. Furthermore, oxaloacetate is converted to glucose as efficiently as pyruvate in liver perfusion experiments [36]. However, the *Pank1* ^−/−^ mice were severely compromised in their response to oxaloacetate or glycerol (Fig. 6E and 6F), suggesting a deficit in NADH and/or ATP, two products provided by fatty acid oxidation during fasting. On the other hand, the knockout animals were competent in ketogenesis and had higher levels of serum ketones after a 48 h fast that was equivalent to the levels in the wild-type animals (Table 1). Although ketone bodies are produced in mitochondria from acetyl-CoA derived from fatty acid oxidation, their production does not require ATP or NADH, two important products of fatty acid oxidation. Formation of ketones also results in the liberation of two CoA molecules. Thus, ketone body formation continued to supply an energy source to the serum and also recycled intracellular CoA, rendering the fast tolerable in the knockout animals. The acetyl-CoA for ketone body formation was provided by the limited fatty acid oxidation in the knockout livers.

## Discussion

Our results demonstrate that PanK1 expression is necessary to maintain a normal intracellular level of CoA and to fully support the metabolic changes during the transition from the fed to the fasted state. The *Pank1* ^−/−^ mice are unable to fully adapt to the metabolic stress of fasting. The CoA-deficient livers have attenuated rates of fatty acid oxidation that are associated with abnormally high accumulation of long-chain acyl-CoAs and acyl-carnitines, and the development of microvesicular steatosis. An approximately 25% deficiency in fatty acid oxidation in the fasted state correlates with accumulation of liver triglyceride over a 48-hour period and a significant impairment of gluconeogenesis from pyruvate, oxaloacetate or glycerol. A marked hypoglycemia results after a 48 h fast of the knockout animals, whereas the serum ketone levels are the same in wild-type and knockout animals following a fast. These data illustrate that ketone body formation is not as dependent on the intracellular CoA level as gluconeogenesis. Unlike glucose formation, ketone synthesis does not require ATP or NADH, both of which are supplied by fatty acid oxidation. The supply of reducing equivalents to the cytosol is one of the key functions of fatty acid oxidation in support of gluconeogenesis [37]. The continued production of ketones renders the fast tolerable, i.e., the animals do not become lethargic or succumb to coma, even when the fast is extended to 72 hours (data not shown).

The acetyl-CoA products of fatty acid oxidation are directed toward ketogenesis or the TCA cycle, and thus all three of these metabolic processes are linked together. The TCA cycle becomes restricted during fasting, however, due to the increase in NADH which inhibits isocitrate dehydrogenase. The oxaloacetate from the TCA cycle is then diverted to gluconeogenesis, thereby sparing acetyl-CoA for ketogenesis [38], [39]. Ketogenesis has the added benefit of releasing two molecules of unesterified (free) CoA for each ketone formed, which can be recycled for additional acetyl-CoA formation from fatty acids in the CoA-deficient liver. Complete oxidation of a 16∶0 fatty acid requires the input of 8 free CoA molecules to produce 8 acetyl-CoAs. In contrast, the TCA cycle utilizes 1 acetyl-CoA which is recycled to succinyl-CoA and then released as 1 free CoA during a complete turn of the cycle. Ketogenesis uses 2 acetyl-CoAs and releases 2 free CoAs. The free CoApool is the largest pool in mammalian cells and it is the free CoA pool that is most reduced in the *Pank1* knockout animals (Fig. 3A).

The phenotype of the PanK1-deficient mouse is reminiscent of humans and animals with mutations in the enzymes of fatty acid oxidation that utilize CoA [17], [40], [41]. The most severely affected are those patients with deficiencies in long-chain acyl-CoA dehydrogenase that catalyzes the first step in the mitochondrial pathway. These subjects exhibit hypoketosis and hypoglycemia during a fast, whereas only fasting hypoglycemia arises from partial defects in β–oxidation [40], [42], [43]. Animals with deficiencies in acyl CoA oxidase [44] or short-chain acyl CoA dehydrogenase [45] exhibit spontaneous steatosis, but animals with deficiencies in very long chain acyl CoA dehydrogenase [46], long chain acyl CoA dehydrogenase [42], medium chain acyl CoA dehydrogenase [47], dienoyl CoA reductase [43], enoyl-CoA isomerase [48] and PPARα [49] exhibit steatosis only upon fasting. Our mice most closely resemble the mitochondrial 2,4-dienoyl-CoA reductase deficient mice [43]. These animals oxidize saturated fatty acids normally, but can only partially degrade unsaturated fatty acids. Like the *Pank1^−/−^* mice, this partial fatty acid oxidation defect gives rise to fasting steatosis and hypoglycemia with normal ketogenesis [43]. Thus, increasing the intracellular level of CoA is as critical to the normal fasting response as the PPARα-mediated up-regulation of the fatty acid oxidation pathway.

A curious aspect of the *Pank1^−/−^* phenotype is the lower triglyceride level in the fed state compared to wild-type animals (Fig. 4), coupled with lower serum triglyceride levels (Table 1). The lower triglyceride accumulation in liver could be due to altered fatty acid uptake, altered fatty acid synthesis or lower triglyceride synthesis, all of which are CoA-dependent processes. Acetylation of the proteins of intermediary metabolism, in particular the enoyl-CoA hydratase/3-hydroxyacyl-CoA dehydrogenase of fatty acid oxidation or the PEP-carboxykinase of gluconeogenesis [50], may also be impacted by lower CoA in the knockout animals, in the fed as well as the fasted state. These regulatory implications are under investigation.

The *Pank1^−/−^* mice recapitulate the abnormal fasting metabolism observed in pantothenate-deficient animals. Fasted rats on a pantothenate-deficient diet are hypoglycemic compared to either ad libitum or pair fed controls [51], [52]. Fasted pantothenate-deficient rats are also impaired in gluconeogenesis [53], while ketogenesis is not affected [54]. Two other consistently documented symptoms in pantothenate-deficient animals are weight loss and neurological problems manifested as spasticity/dragging of the hind limbs or abnormal gait [51], [52], [54]–[56]. Abnormal gait is also observed in pantothenate-deprived humans [57], and are reminiscent of PKAN disease arising from mutations in *PANK2* [58]. However, neither of these symptoms are observed in the *Pank1^−/−^* mice in this study or in the *Pank2^−/−^* mouse model [59]. Thus, the PanK1-deficient mouse exhibits only the metabolic defects associated with global pantothenate deficiency. This conclusion is consistent with the importance of the liver in energy homeostasis during nutrient deprivation and the high PanK1 expression in this organ.

Mitochondrial CoA levels would primarily be affected by lack of PanK1. The rate-limiting enzymes for gluconeogenesis, ketone body formation, fatty acid oxidation and the tricarboxylic acid cycle are all in the mitochondria. It is known that most of the CoA in the cell is in the mitochondria, but CoA biosynthesis occurs in the cytosol. Mitochondria actively import CoA [32] and the CoA concentration in mitochondria is about 100-fold higher than the cytosol [1]. Previously published data show that the ratio of mitochondrial CoA to total CoA is maintained during fasting [20], [60]. Because there is lower total CoA and about the same number of mitochondria (Fig. 3 and 6), we conclude that there is less CoA per mitochondrium in the knockout livers.

Another important discovery in this study is that the regulation of CoA levels during fasting is mediated by factors other than PanK gene expression. The mRNA levels for the PanK isoforms did not increase during fasting. The intracellular activities of PanK2 and PanK3 could be increased by allosteric regulators such as long chain acyl-carnitines [7]; however, the small increase in acyl-carnitine levels in the fasted wild-type animals suggests that this mechanism is not the underlying cause in normal animals. On the other hand, there was substantial elevation in long-chain acyl-carnitines in the CoA-deficient livers, suggesting that stimulation of PanK2 and PanK3 activities may compensate in part for the loss of PanK1. This adaptive mechanism may contribute to the modest increase in CoA in the fasted *Pank1* ^−/−^ animals, as the levels of C16∶0, C18∶0 and C18∶1 acyl-carnitines are significantly elevated. Previous work shows that CoA degradation is substantial in the liver, but not in other tissues [11]. Nudix hydrolases are responsible for CoA degradation. The Nudt7 phosphodiesterase is selectively expressed in liver [24], and the increase in fasting CoA levels correlates with the downregulation of Nudt7 expression [25], [26] (Table 2). Fasting regulation of Nudt7 activity occurs at the level of transcription, and Nudt7 mRNA is strongly downregulated by Wy-14,643, a PPARα agonist [26]. These correlations suggest that Nudt7 has a central role in CoA homeostasis.

The marked effects on fatty acid oxidation due to lower CoA levels may provide some insight into understanding the neurological defects in PKAN disease. PanK1 is expressed at low levels in the brain, which primarily expresses PanK2 and PanK3 [61]. PKAN arises from mutations in the human *PANK2* gene [6], which is localized to the mitochondria in humans [62]. It is not known whether the inactivation of PanK2 results in decreased cellular CoA in human neurons, but the significant effects of PanK1 inactivation in the liver suggest that the elimination of PanK2, a substantial isoform in brain, would have similar consequences for neuronal CoA homeostasis. This reasoning suggests that PanK2 deficiency would also compromise fatty acid metabolism in the brain due to compromised oxidation. This idea suggests that PKAN disease, like Parkinson's [63] and Alzheimer's [64] neurodegenerative diseases, involves defects in mitochondrial function.

## Materials and Methods

### Ethics Statement

All procedures were performed according to protocol 323 which was approved by the St. Jude Children's Research Hospital Institutional Animal Care and Use Committee.

### Generation of *Pank1^−/−^* Mice

The targeting construct (pPJ288) was generated by cloning 3 fragments of mouse *Pank1* genomic DNA into vector pNEOtkLoxP. Plasmid pPJ288 contained a loxP site between a 2.2-kb genomic DNA and the TK and NEO selection cassettes, followed by a second loxP site upstream of a 2-kb genomic DNA fragment containing exon 3, and by a third one upstream of a 3.9-kb genomic DNA (Fig. 1A). The targeting vector was digested with DraIII restriction enzyme prior to transfection into the mouse embryonic stem cell line derived from strain 129/SvEv (Specialty Media), grown on mitotically inactivated mouse embryonic fibroblasts which were resistant to neomycin. Clones resistant to the neomycin analog G418 were selected and screened by PCR using Expand Long Template PCR System (Roche) and two primers: RecF (5′-GGCATAGGCTCCTGAGGC) and T (5′-GGTCCACGACCCAAGCTG). ES cells that had undergone homologous recombination with the targeting construct were identified by a 2456 bp product. The presence of the third loxP site was confirmed by performing PCR with primers Ex3F (5′-GGTCTGAGTGCATTTCTTGTC) and R2 (5′-GCCTAATTTCGTTCACAGTGG) and then digesting the PCR product with BamHI. The wild type allele yielded a product of 1218 bp; the allele with the loxP site yielded two bands of 1072 and 128 bp. Embryonic stem cells containing the recombined *Pank1* DNA at the correct locus were injected into C57BL6/J mouse blastocysts, which were then implanted into pseudopregnant female mice by the St. Jude Transgenic Core Facility. Male chimeric offspring with 75 to 90% agouti color, the coat color contributed by the embryonic stem cells, were bred with C57BL6/J females. Pups that were 100% agouti, indicating germ line transmission, were screened. Tail clips were lysed and multiplex PCR analysis was used to genotype the mice using RED Extract-N-Amp PCR kit (Sigma) and three primers: F (5′-GGATAGGATGGCTACTAGCTGC), R1 (5′-TTACTAGCTAAGTGGCCCAGG) and T (see above). The wild type allele yielded a product of 274 bp, and the recombinant floxed *Pank1* allele yielded a product of 148 bp. FVB/N-Tg(ACTB-cre)2Mrt/J mice expressing Cre recombinase under the human β-actin gene promoter were utilized to generate a deletion within the floxed *Pank1* gene by recombination of the first and third loxP sites, resulting in the loss of exon 3. A multiplex PCR analysis was used to genotype the offspring from the breeding of ACTB-cre mice with mice heterozygous for the floxed *Pank1* using primers F, R1 and R2. A 274-bp product indicated the presence of a wild type allele, and a 220 bp product indicated the presence of a knockout allele. The percentage of C57Bl6 background of the *Pank1* KO and WT mice was between 71 and 74% as measured by genome scanning analysis conducted by Jackson Laboratories. The control mice for the experiment were age matched siblings with the same percentage of C57Bl6 genotype.

### Animal Studies

Unless otherwise stated, 12–16 week-old mice were used for all the experiments. Mice were maintained at a room temperature of 72±2°F, room humidity of 50%±10%, and a 14 hr light, 10 hr dark cycle, with the dark cycle starting at 20:00 hr. For experiments that required fasting, mice were transferred to clean cages containing floor grids and food was removed for the indicated time. The use of floor grids prevented coprophagia during fasting, and reduced the incidence of unusually high glucose levels in mice after a 48 h fast. Water was supplied ad libitum. Glucose levels were measured via tail bleeding using a glucometer (FreeStyle). For the pyruvate, oxaloacetate and glycerol challenges, 4–10 mice per genotype were fasted overnight and then injected intraperitoneally with 2 g/kg of pyruvate or glycerol and 1 g/kg of oxaloacetate (pH = 7.0) in a volume of 10 ml/kg. Glucose was measured before injection of the compounds (t = 0) and after 15, 30, 45, 60, 90 and 180 min. Blood was drawn either by retro-orbital bleeding or by cardiac puncture for triglyceride, free fatty acid and ketone body measurements. Serum triglycerides were analyzed by the Veterinary Pathology Core at St. Jude Children's Research Hospital. Free fatty acids and ketone bodies were determined using the BioVision Fatty Acid Assay Kit and the Stanbio Laboratory β-Hydroxybutyrate Liquicolor kit, respectively, following the manufacturers' instructions. Male mice were used for all procedures which were performed according to protocol 323 approved by the St. Jude Children's Research Hospital Institutional Animal Care and Use Committee.

### Food Monitoring

The mice were fed a regular rodent diet (LabDiet 5013). To determine food intake, mice had unrestricted access to a pre-weighed amount of food, and residual chow was measured every 48 h for 6 days. Food intake was calculated as the difference between food provided and left over, the data were averaged and reported as amount of food eaten per 24 h per mouse. To analyze the lipid balance (lipid intake and output), the lipid content of the feces produced in 24 h was determined gravimetrically in triplicate as described by Argmann *et al.* [65], and subtracted from the total lipids present in the amount of food ingested over 24 h. The results were averaged and reported as % of retained lipids.

### Determination of PanK Expression, PanK Activity and CoA Levels

Mice were euthanized and the livers were quickly excised and either immersed in RNAlater (Ambion) or flash frozen in liquid nitrogen. The PanK isoform expression in knockout and control livers of fed or fasted animals was determined by real time qRT-PCR using the primers and probes previously reported [61]. Primers for PGC1α, PEPCK, G6Pase, Nudt7 and Nudt19 are listed in Table 3. To determine the PanK activity, ∼200 mg of frozen tissue was homogenized in cold buffer (1 ml, 20 mM K_2_HPO_4_, 1 mM ATP, pH 7.4) using a Dounce tissue grinder. The homogenates were centrifuged at 20,000×g, 4°C for 45 min, and the supernatants were dialyzed overnight in the homogenization buffer. The protein concentration of the dialyzed samples was determined [66] and adjusted to 20 mg/ml in 20 mM K_2_HPO_4_, pH 7.4. PanK activity was assayed in reaction mixtures containing 100 mM Tris-HCl, pH 7.4, 10 mM MgCl_2_, 2.5 mM ATP, 90 µM d-[1-^14^C]pantothenate (specific activity, 27.5 mCi/mmol), 0–200 µg of homogenate protein. Samples were incubated at 37°C for 30 min, after which time the reactions were stopped and analyzed as previously described [4]. The inhibitory effect of acetyl-CoA on the liver PanK activity was determined by including increasing concentrations of the compound in the reaction mixtures, as indicated. The free CoA and CoA thioester levels were determined as previously described [61].

**Table 3:** List of primers used for quantitative real time PCR.

*Gene*	*Forward Primer (5′→3′)*	*Reverse Primer (5′→3′)*
*Nudt7*	CCAAGTGGAGGTGGTCTCTC	GATGAAATCACGGCCAGACT
*Nudt19*	ATCTGTGCCATCCGCGAAGC	CACAGCTGGAGGAAGCAGCG
*Ppargc1a*	TAGGCCCAGGTACGACAGC	GCTCTTTGCGGTATTCATCC
*Pck1*	GCTGGCAGCATGGGGTGTTT	TTGGGCAACTTGGCTGCTGG
*G6pc*	CTTTCAGCCACATCCGGGGC	CCCATTCTGGCCGCTCACAC

### Determination of Mitochondrial Number

Mice (4–5 per genotype) were sacrificed in the fed state or following a 48h fast for mitochondrial DNA quantitation. The livers were quickly removed, snap frozen in liquid nitrogen and stored at −80°C until used. Genomic DNA was extracted from these samples using the protocol described by Strauss [67]. Relative mitochondrial copy number to nuclear copy number was determined by RT-PCR as described by Stites et al. [68] using 150 ng of DNA for nuclear DNA quantitation and 400 pg for mitochondrial DNA quantitation. Each sample was analyzed in quadruplicate.

### Lipid Analysis

Lipids were extracted from ∼50 mg of liver using a modification of the Bligh and Dyer procedure [69] optimized for lipid quantitation by the LipidMaps group [70]. The amount of each major lipid class was estimated by flame-ionization thin layer chromatography using an Iatroscan MK-5 (Iatron, Tokyo, Japan). Triglycerides, cholesterol and cholesterol esters were separated using hexane∶ether (90/10, v/v). Phospholipids were resolved using chloroform∶methanol∶acetic acid∶water (50/25/8/2, v/v/v/v). The lipids were identified by co-migration with authentic standards and quantified by comparison with known amounts of these standard lipids.

### Histology and Electron Microscopy

Hematoxylin and eosin (H&E) and Oil Red O (ORO) staining of liver sections was performed by the Veterinary Pathology Core at St. Jude Children's Research Hospital. Analysis by Transmission Electon Microscopy was performed by the Cell and Tissue Imaging Core at St. Jude Children's Research Hospital. For H&E staining, the livers were fixed in 10% formalin. ORO staining was performed on frozen sections embedded in Shandon Cryomatrix (Thermo Scientific). Animals were perfused with EM grade 4% paraformaldehyde (Electron Microscopy Sciences) [71], the liver excised and stored in 2.5% glutaraldehyde in 0.1 M sodium cacodylate buffer at 4°C for 24h before processing. The tissue was then rinsed in the same buffer 3 times and dehydrated in a graded series of alcohol and then propylene oxide. The tissue was infiltrated and embedded in epon araldite and polymerized overnight at 70°C. The 70 nm sections were cut on a Lieca UC6 ultramicratome using a Diatome diamond knife. They were stained with lead citrate and 8% uranyl acetate. The sections were imaged on a JEOL 1200EX11 Transmission Electron Microscope with an AMT XR111 11 megapixel Digital Camera.

### Measurements of Fatty Acid Oxidation

The procedure described by Adams *et al.* [72] was adapted to measure the rate of [9,10-^3^H]palmitic acid oxidation in the liver homogenates of *Pank1* knockout and control mice. Duplicate samples were used. Briefly, livers from *Pank1* knockout and control mice were excised, rinsed in cold isolation buffer (220 mM mannitol, 70 mM sucrose, 2 mM Hepes, 0.1 mM EDTA, pH 7.2), blotted dry, pooled (2–3 per group) and weighed. The livers were homogenized in isolation buffer to a final concentration of 400 mg of tissue/ml. The reaction buffer contained 1 mM L-carnitine, 13.1 mM sucrose, 78 mM Tris-HCl, 10.5 mM K_2_HPO_4_, 31.5 mM KCl, 5 mM ATP, 1 mM NAD^+^, 850 µM EDTA, 500 µM palmitic acid, 1.7 µM of [9,10-^3^H]palmitic acid (60 Ci/mmol) and 100 mg/ml fatty acid-free BSA, pH 7.4. Ethanol stock solutions of radiolabeled and unlabeled palmitic acid were added directly to the reaction buffer. Reactions were started by the addition of 100 µl of homogenate to 250 µl of reaction buffer and incubated at 37°C for 15 min. The reactions were stopped by adding 150 µl of 30% perchloric acid and centrifuged at 20,000×g, 4°C for 2 min to pellet the precipitated proteins. The supernatants were set aside and the pellets washed with 0.5 ml of water and centrifuged again. The two supernatants per sample were then combined and the [^3^H]water produced by β-oxidation in each sample was separated from [9,10-^3^H]palmitic acid using the lipid extraction procedure described by Bligh and Dyer [69]. Aliquots of the aqueous phases were transferred to scintillation vials and counted.

### Quantification of Acyl-Carnitines

Acyl-carnitines were extracted, identified and quantified by mass spectrometry as described previously [73], [74]. Acyl-carnitines were extracted from 250 mg of liver by freezing the sample in liquid nitrogen and grinding the sample into a powder. To the liver powder, 1 ml of 8∶2 (v/v) acetonitrile∶water was added along with 70 µl of Labeled Carnitine Standards Set B (Cambridge Isotope Laboratories, Inc.). The suspension was sonicated six times for 10 s using the Sonicator 3000 (Misonix). The samples were centrifuged at 16000×g for 5 min and the supernatant was saved. The supernatant was added to a Bond Elut – PRS column (Varian) which was activated by washing the column with 3 ml of methanol and 3 ml 100∶3.5 (v/v) methanol∶HCl followed by two 3 ml washes of water. The column was again washed twice with 3 ml water and once with 3 ml methanol. Elution from the column was done three times with 1 ml of 40 mM BaCl_2_ in 75% methanol. Elution fractions were pooled together and evaporated under nitrogen. The residue was resuspended in 3 ml acetonitrile and transferred to a new tube to be evaporated. To the residue, 200 µl of 4∶1 (v/v) 2-propanol∶acetylchloride was added and incubated a 65°C for 15 min. The reaction mix was evaporated under nitrogen, and 300 µl of acetonitrile was added to resuspend the sample. This resuspension was used to measure the acyl-carnitines by taking 70 µl of the sample and adding 30 µl of water. This dilution was used for direct-infusion ESI-MS technology by the Hartwell Center for Bioinformatics and Biotechnology, St. Jude Children's Research Hospital using a Finnigan™ TSQ® Quantum (Thermo Electron, San Jose, CA) triple quadrupole mass spectrometer. The instrument was operated in the positive ion mode with the following ion source parameters: spray voltage 3500 V, capillary temperature 120°C, capillary offset 35 V, and tube lens offset was set by infusion of the polytyrosine tuning and calibration solution (Thermo Electron, San Jose, CA) in electrospray mode. Acquisition parameters for propylated acylcarnitines were: scan range 180–600 *m/z*, scan time 0.5 s, product mass 85 *m/z*, collision energy 20 V, peak width Q1 and Q3 0.7 FWHM, and Q2 CID gas 0.5 mTorr. To calculate the amount of each acyl-carnitine, the peak height for each acyl-carnitine was compared to the peak height of the appropriate internal standard.
